# Thermal Properties and Flammability Characteristics of a Series of DGEBA-Based Thermosets Loaded with a Novel Bisphenol Containing DOPO and Phenylphosphonate Units

**DOI:** 10.3390/ma15217829

**Published:** 2022-11-06

**Authors:** Corneliu Hamciuc, Tăchiță Vlad-Bubulac, Diana Serbezeanu, Ana-Maria Macsim, Gabriela Lisa, Ion Anghel, Ioana-Emilia Şofran

**Affiliations:** 1 Department of Polycondensation and Thermally Stable Polymers, “Petru Poni” Institute of Macromolecular Chemistry, 41A, Grigore Ghica Voda Alley, 700487 Iasi, Romania; 2 Department of Chemical Engineering, Faculty of Chemical Engineering and Environmental Protection, “Gheorghe Asachi” Technical University of Iasi, Bd. Mangeron 73, 700050 Iasi, Romania; 3 Fire Officers Faculty, Police Academy “Alexandru Ioan Cuza”, Morarilor Str. 3, Sector 2, 022451 Bucharest, Romania

**Keywords:** epoxy thermosets, phosphorus-containing bisphenol, thermal stability, flame resistant

## Abstract

Despite a recent sustained preoccupation for developing biobased epoxies with enhanced applicability, such products have not been widely accepted for industry because of their inferior characteristics compared to classic petroleum-based epoxy thermosets. Therefore, significant effort is being made to improve the flame retardance of the most commonly used epoxies, such as diglycidyl ether-based bisphenol A (DGEBA), bisphenol F (DGEBF), novalac epoxy, and others, while continuously avoiding the use of hazardous halogen-containing flame retardants. Herein, a phosphorus-containing bisphenol, bis(4-(((4-hydroxyphenyl)amino)(6-oxido-6H-dibenzo[c,e][1,2]oxaphosphinin-6-yl)methyl)phenyl) phenylphosphonate (BPH), was synthesized by reacting bis(4-formylphenyl)phenylphosphonate with 4-hydroxybenzaldehyde followed by the addition of 9,10-dihydro-9-oxa-10-phosphaphenanthrene-10-oxide (DOPO) to the resulting azomethine groups. Environmentally friendly epoxy-based polymer thermosets were prepared by using epoxy resin as polymer matrix and a mixture of BPH and 4,4′-diaminodiphenylsulfone (DDS) as hardeners. A hyperbranched phthalocyanine polymer (HPc) and BaTiO_3_ nanoparticles were incorporated into epoxy resin to improve the characteristics of the final products. The structure and morphology of epoxy thermosets were evaluated by infrared spectroscopy and scanning electron microscopy (SEM), while the flammability characteristics were evaluated by microscale combustion calorimetry. Thermal properties were determined by thermogravimetric analysis and differential scanning calorimetry. The surface morphology of the char residues obtained by pyrolysis was studied by SEM analysis.

## 1. Introduction

Epoxy resins, an important class of thermosetting resins, are widely applied in high-performance engineering fields because of their useful characteristics such as good electrical, thermal, and mechanical resistance, good adhesion to many substrates, resistance to solvents and chemicals, and low toxicity [1,2,3]. A major disadvantage of epoxy resins is their high flammability, which restricts their utility in various applications in the construction, transportation, aerospace, electric, and electronic fields. Therefore, much research has been undertaken to improve their flame resistance [4,5,6]. An effective procedure to decrease the flammability of epoxy resins consisted in the recent past of the use of flame retardants containing halogen. However, the resulting materials turned out to be extremely dangerous because when burning they produce very toxic substances and a large amount of heat and smoke [7,8].

Recently, research efforts have been undertaken to obtain environmentally friendly flame retardants, such as those containing phosphorus. They attract much interest because of their high efficiency in reducing the flammability of the polymers, as well as the fact that they are nontoxic and do not produce very toxic substances while burning [9,10].

Flame retardant utilized for epoxy resins can be divided into two main categories, additive and reactive. The additive flame retardants are incorporated and dispersed into epoxy resin; this method is convenient in terms of application possibilities and effective cost. However, a higher concentration of additive flame retardant can lead to a deterioration of the final properties of the thermosets; also, the risk of leaching of the flame retardant from the polymer is an important drawback [11]. A noticeable advantage of reactive phosphorus-containing flame retardants is represented by the fact that they can be either introduced into epoxy resin structure, as a part of the macromolecular architecture, or they can be used as crosslinking agents to obtain high-performance epoxy thermosets [12].

Among the flame retardants that contain phosphorus, phosphonates and polyphosphonates are largely applied to reduce the flammability of polymers [13,14,15]. These compounds are used as additive flame retardants or are introduced into the polymer structure during synthesis and act both in the gas and condensed phases. An important group of phosphorus-containing flame retardants is represented by 9,10-dihydro-9-oxa-10-phosphaphenanthrene-10-oxide (DOPO) and its derivatives. DOPO has a heterocyclic structure and exhibits good thermal and chemical stability. It has an active C-H bond and can react with a variety of compounds that contain double bonds, imine, epoxy, or carbonyl groups, yielding a diverse range of phosphorus flame retardants [16]. However, a high content of DOPO groups is required to achieve a significant improvement in the flame resistance of epoxy resins, which can lead to a deterioration of the end-use product’s characteristics. Therefore, the DOPO monomer has been frequently appealed in the synthesis of various flame retardants containing nitrogen, sulfur, silicon, or boron in the structure, elements well-known for their synergistic effect in terms of flame retardancy when used along with phosphorus, thus, showing better flame retardancy in epoxy-resin-based materials [15,17,18,19,20,21,22,23]. Various diamines and bisphenols containing phosphorus and nitrogen as reactive flame retardants for crosslinking epoxy resin have been reported [24,25,26,27,28,29]. Xiong et al. reported the preparation of two bisphenols by interacting DOPO with the adducts of 4-hydroxybenzaldehyde with 4-aminophenol and 4,4′-diaminodiphenylsulfone, respectively. These compounds were further used as co-curing agents for epoxy resins, resulting in epoxy thermosets with excellent thermal stability, high glass transition temperature, and improved flame resistance [24]. Lin et al. conducted similar work involving the addition of DOPO to a preformed imine group of some bisphenols [25]. Their reported epoxy thermosets exhibited good flame retardancy and moderate glass transition temperature and thermal stability. The presence of segments coming from another bisphenol, namely 2-(phenylphosphoryl)-1,4-benzenediol, considerably decreased the combustion parameters (heat release rate (HRR) and total heat release (THR)) measured by cone calorimetry, as Zhang et al. noted [26]. The group of Zhao et al. developed an aromatic diamine having two DOPO groups and a phenylphosphonate unit and used it as a co-curing agent to prepare epoxy thermosets, leading to materials with good flame retardancy, mechanical properties, and high glass transition temperature. The incorporation of only 2 wt% diamine improved the limiting oxygen index (LOI) up to 31% and the sample achieved self-extinguishing capacity (UL-94 V-0 rating) [27].

Metal phthalocyanines are a type of macrocyclic compounds with a conjugated system and a high nitrogen atom content that can help to improve polymer flame resistance and thermal stability. They have been widely used in many different areas, such as semiconductors, catalytic reactions, gas sensors, high dielectric materials and solar cells [30,31]. Hyperbranched phthalocyanine polymers are useful as additives to improve thermal properties and flame resistance; also, they can be used together with other flame retardants, such as those containing phosphorus, to achieve a flame retardant synergistic effect. A hyperbranched copper phthalocyanine compound (HPc) was synthesized and used for the preparation of polyarylene ether nitriles/HPc hybrid films showing high thermal stability and a high dielectric constant [32]. A hyperbranched phthalocyanine polymer with phosphorus was produced as flame retardant to reduce the flammability of epoxy resins. The incorporation of 15 wt% of this additive into epoxy resin led to a material that passed the UL-94 V-0 rating showing an LOI of 35.8% [33]. Because of its high dielectric permittivity values and temperature-dependent polarization, BaTiO_3_ is regarded as a very promising filler capable of improving the dielectric properties and functionality of polymer hosts. As a result, certain research groups recently prepared and reported nanocomposites with appealing dielectric, energy storage, and charge transport properties based on various epoxies doped with varying volume ratios of BaTiO_3_ nanosized particles [34,35,36].

In this paper, a phosphorus-containing bisphenol BPH was prepared by addition of DOPO monomer to the imine groups of a compound resulting from the condensation reaction of a phenylphosphonic dialdehyde with p-aminophenol. BPH was used as a co-curing agent to improve the flame resistance of epoxy resin. Additionally, a hyperbranched copper phthalocyanine polymer (HPc) and BaTiO_3_ nanoparticles were incorporated into epoxy resins to increase the flame resistance, thermal stability, and dielectric properties of the resulting epoxy composites. The flame resistance of epoxy thermosets, investigated by microscale combustion calorimetry, was significantly improved by using BPH as co-curing agent and by incorporation of HPc into epoxy resin.

## 2. Materials and Methods

### 2.1. Materials

**Figure 1:**
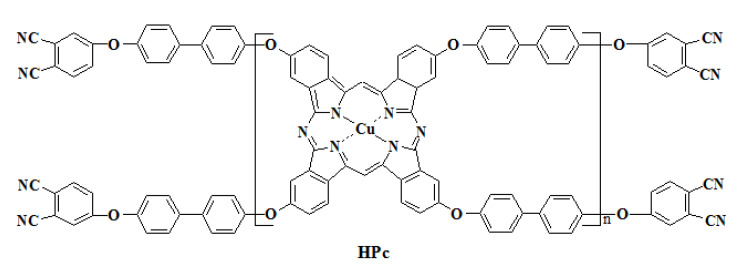
Chemical structure of hyperbranched copper phthalocyanine polymer (HPc).

9,10-Dihydro-9-oxa-10-phosphaphenanthrene-10-oxide (DOPO) was purchased from Chemos GmbH, Germany, and it was freshly dehydrated before use. Phenylphosphonic dichloride, 4-hydroxybenzaldehyde, 4-aminophenol, *N,N*-dimethylformamide (DMF), triethylamine (Et_3_N), BaTiO_3_ nanoparticles (cubic crystalline phase, <100 nm particle size (BET)), and 4,4′-diphenylsulfone (DDS) were provided by Sigma-Aldrich and used as received. Epoxy resin based on bisphenol A diglycidyl ether, with an epoxy equivalent weight (EEW) of 0.53 equiv. per 100 g (Mn~377 g/mol), was purchased from Sigma Aldrich. A hyperbranched Cu phthalocyanine polymer (HPc) was synthesized starting from 4,4′-bis(3,4-dicyanophenoxy)biphenyl and CuCl, following a method adapted from a previously reported procedure [32]. The chemical structure of HPc is shown in Figure 1.

Aromatic dialdehyde, bis(4-formylphenyl) phenylphosphonate, was prepared by the reaction of phenyl phosphonic dichloride and 4-hydroxybenzaldehyde according to a method previously described [27].

### 2.2. Synthesis of Bis(4-(((4-hydroxyphenyl)amino)(6-oxido-6H-dibenzo[c,e][1,2] Oxaphosphinin-6-yl)methyl)phenyl) Phenylphosphonate (BPH)

**Figure 2:**
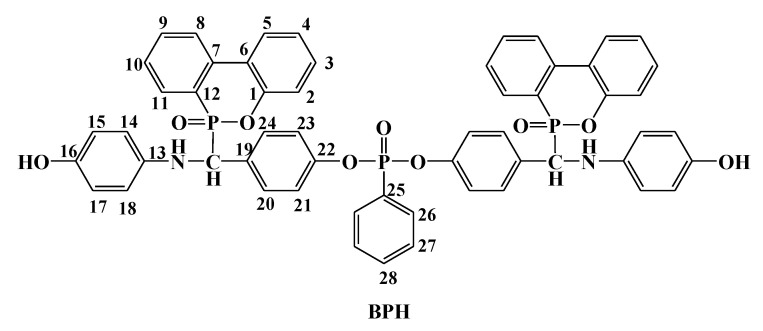
Chemical structure of BPH bisphenol.

Bis(4-formylphenyl) phenylphosphonate (3.66 g, 0.01 mol), 4-aminophenol (2.18 g, 0.02 mol), and 30 mL ethanol were introduced into a three-necked flask equipped with a condenser and magnetic stirrer. The reaction mixture was stirred at reflux temperature under nitrogen atmosphere for 5 h. DOPO (4.32 g, 0.02 mmol) and 8 mL ethanol were introduced into the flask and heating was continued at reflux temperature for 18 h. To yield 85% of the final product, BPH (Figure 2), the resulting precipitate was filtered, washed with water for several times, and dried under vacuum at 50 °C for 5 h.

FTIR (KBr, cm^−1^): 3070 (aromatic C–H), 1596 and 1508 (aromatic –C=C–), 1464, 1373 (C–N), 1200 (P=O groups), 815, 753 (Figure S1, Supporting Information).

^1^H NMR (600.13 MHz, DMSO-*d_6_*, ppm): 4.99–5.03 (1H, m, CH one isomer), 5.43–5.47 (0.8H, m, OH second isomer), 5.67–5.69 (0.8H, m, NH one isomer), 6.1–6.12 (1H, m, NH, second isomer), 6.40–6.50 (7.3H, dd, J_H-P_ = 9.1 Hz, 9.1 Hz, H14, H15, H17, H18 both isomers), 6.98 (3.5H, t, J_H-P_ = 6.9 Hz, H2, one isomer), 7.08–7.13 (3.5H, m, H21, H23 both isomers), 7.20 (0.9H, d, J_H-P_ = 8.3 Hz, H2, second isomer), 7.30 (1.8H, t, J_H-P_ = 7.5 Hz, H4, both isomers), 7.38–7.45 (6.1H, m, H20, H24, H3 both isomers), 7.56 (1H, t, J_H-P_ = 7.5 Hz, H10, both isomers), 7.60–7.64 (1.8H, m, H27, both isomers), 7.70–7.75 (2.4H, m, H28, both isomers), 7.80 (1H, t, J_H-P_ = 7.9 Hz, H9, both isomers), 7.92–7.95 (1.8H, m, H26, both isomers), 8.01–8.04 (1H, m, H11, both isomers), 8.13–8.20 (3.6H, m, H8, H5, both isomers), 8.50–8.53 (1.8H, s, OH, both isomers) (Figure S2).

^13^C NMR (100.61 MHz, DMSO-*d_6_,* ppm): 54.92–57.09 (m, CH), 115.3 (d, J_C-P_ = 6.6 Hz, C14, C15, C17, C18), 119.9 (s, C21, C23), 120.3–119.9 (m, C2), 121.4 (m, C6), 122.6 (d, J_C-P_ = 39.4 Hz, C7), 124.06–123.8 (m, C8), 124.7 (d, J_C-P_ = 11.2 Hz, C4), 125.6 (d, J_C-P_ = 26.2 Hz, C5), 128.45–128.10 (m, C10), 129.1 (d, J_C-P_ = 15.2 Hz, C27), 129.1 (d, J_C-P_ = 16.6 Hz, C20, C24, 28), 130.7 (d, J_C-P_ = 24.9 Hz, C3), 131.7 (d, J_C-P_ = 8.9 Hz, C11), 131.9 (d, J_C-P_ = 9.8 Hz, C26), 132.5 (d, J_C-P_ = 16.7 Hz, C19), 133.6 (d, J_C-P_ = 43.1 Hz, C25, C9), 135.3 (m, C12), 139.1 (m, C13), 148.7 (m, C1), 149.3 (m, C22), 149.4 (s, C16) (Figure S3).

^31^P NMR (242.94 MHz, DMSO-*d_6_*, ppm): 28.5–31.4 P in DOPO units, 11.9 P in phenylphoshonate units (Figure S4).

### 2.3. Preparation of Epoxy Resin Nanocomposites

Epoxy resin was cured using mixtures of BPH and DDS as hardeners. The formulations of the pre-curing mixtures of epoxy resin, BPH, HPc, and BaTiO_3_ are listed in Table 1. The molar ratio of reactive “H”, in both DDS and BPH reactive additives utilized as hardeners, to the oxirane units of the DGEBA-based resin was approximately 1:1 in each sample. To prepare the EP-1 and EP-2 samples, epoxy resin was mixed with BPH to obtain a translucent mixture; then, DDS was added and the resulting system was heated, under vigorous stirring, up to 130 °C to obtain a homogeneous composite mixture. The product was poured into aluminum molds and then thermally cured at 120 °C for 6 h and at 140, 160, 180, and 200 °C, for 1 h at each temperature. The resulting thermosets were cooled slowly to room temperature to prevent cracking. For EP-3, the above procedure was replicated, with the addition of a solution of HPc in DMF followed by the heating at 80 °C for 2 h before introduction of DDS into the system. In the case of EP-4 and EP-5, epoxy resin was first mixed with BaTiO_3_ nanoparticles and ultrasonication was performed for 30 min at 50 °C, following the procedure explained for the preparation of EP-1 and EP-2.

### 2.4. Measurements

The structure of BPH was investigated by using FTIR and NMR spectroscopy. The FTIR spectrum was recorded on a FTIR Bruker Vertex 70 Spectrophotometer. The structure of epoxy thermosets was investigated by FTIR spectroscopy using a BioRad ‘FTS 135′ FTIR spectrometer equipped with a Specac “Golden Gate” ATR accessory. A LUMOS microscope Fourier transform infrared (FTIR) spectrophotometer (Bruker Optik GmbH, Ettlingen, Germany), equipped with an attenuated total reflection (ATR) device, was used to record the scans between 4000 and 600 cm^−1^ at a resolution of 4 cm^−1^.

^1^H, ^13^C, and ^31^P NMR spectra were recorded on a Bruker Avance Neo 400 MHz and 600 MHz at 400.13 MHz and 600.13 MHz for ^1^H, 100.61 MHz for ^13^C, and 242.94 MHz for ^31^P. ^1^H and ^13^C NMR chemical shifts (δ) in ppm were calibrated to the residual solvent peaks (DMSO-d6, 2.512 ppm for ^1^H and 39.4761 ppm for ^13^C).

Microscopic investigations of epoxy thermosets and of their corresponding chars were performed on an environmental scanning electron microscope, type Quanta 200, operating at 10 kV with secondary electrons in low-vacuum mode (LFD detector). The Quanta 200 microscope is equipped with an energy dispersive X-ray (EDX) system for qualitative and quantitative analysis and elemental mapping.

Thermogravimetric (TG) curves and thermogravimetric derivative (DTG) curves of BPH and epoxy thermosets were recorded with a Mettler Toledo TGA-SDTA851^e^ equipment (Mettler Toledo, Greifensee, Switzerland), in nitrogen atmosphere, and a heating rate of 10 °C/min, in the temperature range of 25–700 °C. The mass of the tested samples was 3.21 ± 0.83 mg. An alumina crucible with lid with a capacity of 70 μL was used. The thermogravimetric (TG) and derivative thermogravimetric (DTG) curves were processed with Mettler Toledo STAR^e^ software (Version 9.10 (Giessen, Germany)). The reproducibility of the TG/DTG curves was verified by recording several tests for each sample under the same conditions and differences of less than 1% were found.

Differential scanning calorimetry (DSC) measurements of BPH and epoxy thermosets were carried out using a Mettler Toledo DSC1-type device (Mettler Toledo, Greifensee, Switzerland) in an inert atmosphere (nitrogen), with a heating rate of 10 °C/min and nitrogen purge at 100 mL/min. The samples with masses of 5.39 ± 1.37 mg have been encapsulated in aluminum crucibles with a capacity of 40 μL. The DSC measurements included three scans (heating–cooling–heating) performed in the temperature range of 25–250 °C with a heating/cooling rate of 10 °C/min. The recorded DSC curves were also processed with the STAR^e^ software (Version 9.10 (Giessen, Germany)) from Mettler Toledo.

The flammability of samples in controlled temperature circumstances was evaluated using microscale combustion calorimetry (MCC) experiments; the temperature in the combustor was 900 °C, and the pyrolyzer was heated up to 750 °C at a rate of 1 °C/s. The tests were carried out in accordance with “Method A” (ASTM D7309-13) [37].

## 3. Results and Discussion

### 3.1. Synthesis and Characterization of BPH

**Scheme 1:**
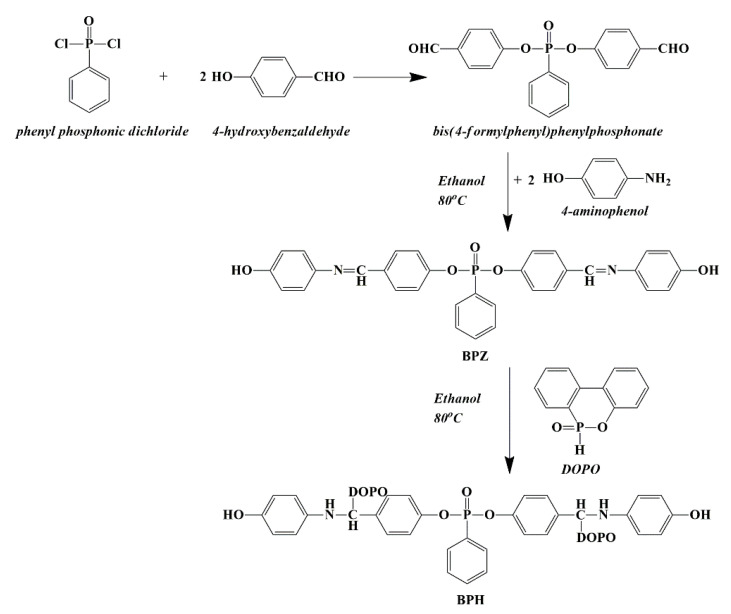
Synthetic pathway to prepare BPH bisphenol.

BPH was prepared in three steps. In the first step, an aromatic dialdehyde, bis(4-formylphenyl) phenylphosphonate, was prepared by condensation reaction of phenyl phosphonic dichloride with 4-hydroxybenzaldehyde. In the next step, the resulting dialdehyde was reacted with 4-aminophenol to yield a bisphenol containing two preformed azomethine groups (BPZ), which was treated in situ with DOPO in the third step of the synthetic pathway to obtain the phosphorus-containing bisphenol denoted as BPH (Scheme 1).

The structure of BPH was determined by FTIR and NMR spectroscopy. In the BPH FTIR spectrum, characteristic absorption bands appeared at 3070 cm^−1^ (aromatic C–H), 1600 and 1596 cm^−1^ (aromatic –C=C–), 1373 cm^−1^ (C–N), 1200 cm^−1^ (P=O groups), 928 cm^−1^ (P–O–Ar link), 753 cm^−1^ (due to the deformation vibration caused by 1,2-disubstituted aromatic DOPO rings), and 815 cm^−1^ (deformation of *p*-phenylene rings) (Figure S1, Supplementary Information).

The structure of BPH was also characterized by ^1^H NMR, ^13^C NMR, and ^31^P NMR. The NMR analysis of BPH showed the existence of two diastereomers in a molar ratio of 1:0.8. The presence of the phosphorus atoms in the structure complicates the shape of the signals in the NMR spectra. This phenomenon has been reported in the literature as attributed to the chirality of the phosphorus stereocenter of DOPO [38].

The ^1^H NMR spectrum of BPH displays some characteristic signals: the hydroxyl group has a broad resonance signal in the region 8.50–8.53 ppm, the CH group proton was associated with the two multiplets (one for each isomer) from 4.99 ppm and 5.47 ppm, the NH group proton was associated with the two multiplets (one for each isomer) from 5.67 ppm and 6.12 ppm, and all aromatic protons have complex resonance signals in the region 6.38–8.20 ppm, mainly because of the proton–phosphorus couplings, and also because of the presence of the two isomers causing more overlap of the signals. The aromatic protons most shielded are *para*-substituted of phenyl, with the signals in the interval 6.38–6.54 ppm (Figure S2).

In the carbon spectrum, all the signals appear as doublets or multiplets because of carbon-phosphorus coupling and signals overlap. As can be seen in Figure S3, the CH group has multiplets at 57 ppm and 60 ppm, while the signals for the aromatic carbon atoms appear in the range 119 ppm to 149 ppm, the quaternary carbon directly linked to oxygen being the most de-shielded in the interval 149.4–148.7 ppm.

The phosphorus spectrum supports the existence of the two isomers through the doublet signals for each isomer; it is clearly observed for the two phosphorus atoms in DOPO (31.39 and 28.47 ppm). For the P-linked phenyl group the two isomers overlap and there is one signal at 11.97 ppm (Figure S4).

The thermal transitions of BPH were determined by DSC measurements (Figure S5). In the first heating run, an amorphous-to-glassy thermal transition was evidenced by the endothermic peak centered at 136 °C, which may correspond to the relaxation enthalpy of physical ageing, which is a quite common occurrence for organic glasses. This assumption was clarified by introspecting the DSC trace of the first cooling run (Figure S5, blue curve), as the sigmoidal alure is suggestive for a vitrification tendency of the glassy state of BPH under cooling from temperatures above 160 °C. In the second heating run, the DSC trace exhibited a similar feature as in the first heating run, revealing the same amorphous-to-glassy state thermal transition. The thermal stability of BPH was investigated by thermogravimetric analysis. The monomer decomposed in several steps. A decrease in the decomposition rate appeared in the temperature range 550–700 °C, suggesting the formation of a more thermostable residue.

### 3.2. Structural and Morphological Characterization of Epoxy-Based Nanocomposites

Cured epoxy composites were prepared by using BPH as a co-curing agent with DDS and incorporating HPc and BaTiO_3_. The amount of BPH in epoxy composites was calculated to have a content of 1 or 2 wt% phosphorus. Two samples were prepared by introducing additionally 10 or 20 wt% BaTiO_3_ nanoparticles. The HPc content was 10 wt%.

**Figure 3:**
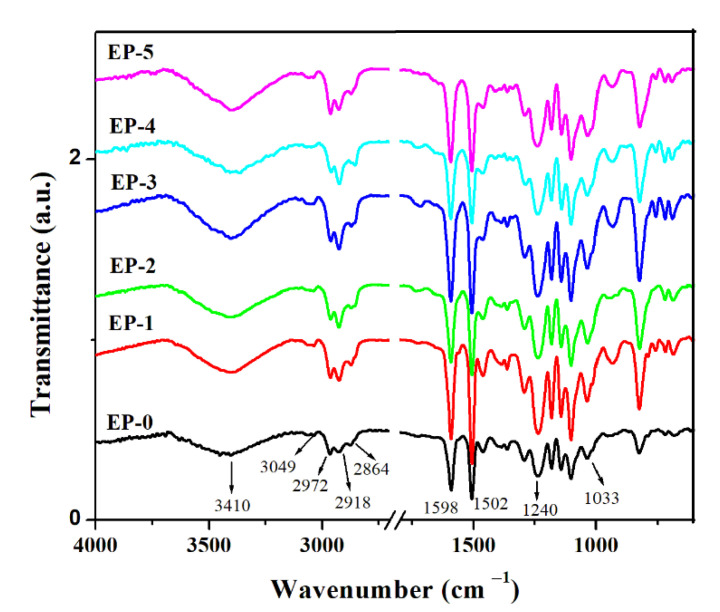
FTIR spectra of epoxy thermosets.

FTIR spectroscopy was used to investigate the structure of epoxy thermosets. In the FTIR spectra of the samples (Figure 3), characteristic absorption bands appeared at 3700–3100 cm^−1^ (N–H and O–H resulting from the crosslinking reaction of epoxy resin with the hardeners DDS and BPH), 3049 cm^−1^ (C–H aromatic), 2972, 2818 and 2864 cm^−1^ (aliphatic stretching vibration), 1598 and 1502 cm^−1^ (aromatic –C=C– stretching vibration), and 1240 and 1033 cm^−1^ (C_6_H_4_–O–CH_2_ asymmetric and symmetric stretching vibration, respectively).

**Figure 4:**
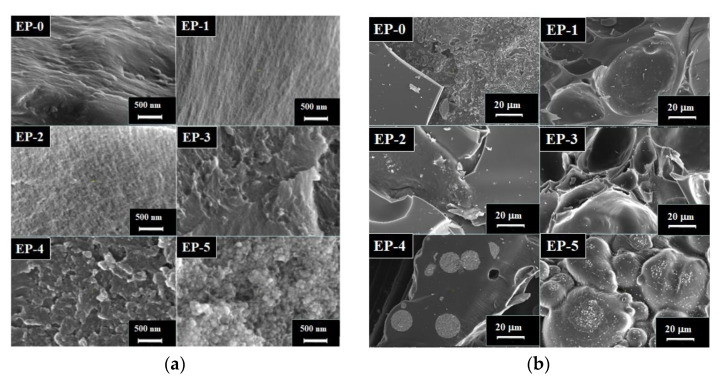
(**a**) SEM micrographs of as synthesized epoxy thermoset surfaces; (**b**) SEM micrographs of epoxy thermosets heated up to 700 °C, with the heating rate of 10 °C/min, under nitrogen atmosphere.

Figure 4a shows the SEM images of the samples. EP-0 fracture surface was smooth, suggesting a glassy and homogeneous microstructure. A modification of the fracture surface was observed by using BPH as co-curing agent. Thus, the EP-1 and especially EP-2 fracture surfaces exhibited higher roughness. The segments coming from BPH were distributed homogenously and agglomerations or phase separation were not observed. In the case of EP-3, the presence of HPc led to a fracture surface with higher roughness. SEM micrographs of EP-4 and EP-5 showed that BaTiO_3_ nanoparticles were relatively homogeneously dispersed within epoxy resin.

**Figure 5:**
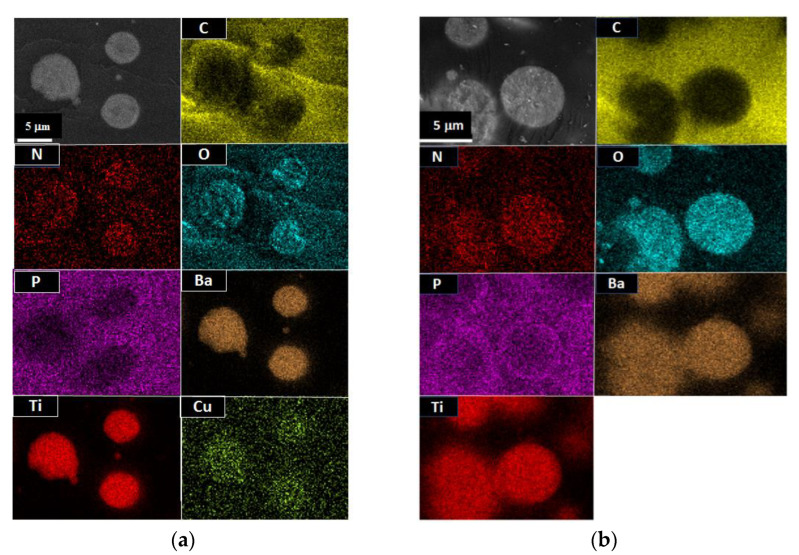
(**a**) EDX mapping of EP-4; (**b**) SEM micrographs of epoxy thermosets heated up to 700 °C, with the heating rate of 10 °C/min, under nitrogen atmosphere.

**Figure 6:**
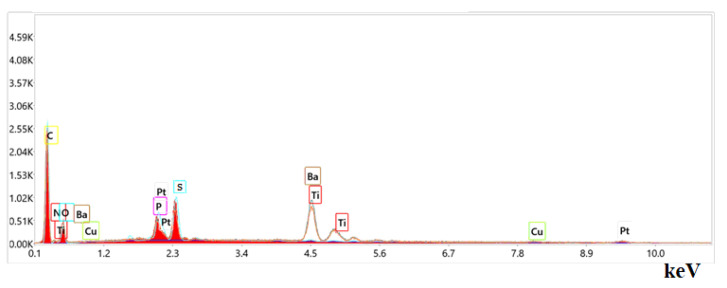
EDX of EP-4.

The EDX mapping of EP-3 (Figure S7) revealed the distribution of phosphorus and Cu atoms, suggesting a uniform distribution of the segments coming from BPH and a homogenous distribution and a good compatibility of hyperbranched phthalocyanine polymer HPc with epoxy matrix. The EDX mapping of EP-4 (Figure 5a) shows the uniform distribution of phosphorus and Cu atoms. The distribution of Ba and Ti atoms in the epoxy matrix, however, suggested that some agglomeration of BaTiO_3_ nanoparticles appeared. In the EDX diagram of EP-4 (Figure 6), the peak at 4.5 KeV revealed the existence of barium and titanium atoms. The presence of sulfur, phosphorus, and copper atoms can be also observed in the EDX diagram.

### 3.3. Thermal Characterization of Nanocomposites

Differential scanning calorimetry measurements were performed to obtain information regarding the expected glass transitions of the epoxy thermosets. All the DSC curves exhibited classical *T_g_*’s, with decreasing values, as the composite systems became more complicated (Table 2, Figure S5b). The *T_g_* values were situated in the temperature range of 158–201 °C. The EP-0 exhibited the highest *T_g_* value of 201 °C. This is because EP-0 does not contain additives and the cross-linking density is high. The use as hardeners of BPH together with DDS decreased the *T_g_* values, especially for EP-2, probably because of the bulkiness of the molecule causing steric hindrance for formation of the network. The presence of HPc and BaTiO_3_ decreased the *T_g_* values when compared with EP-1. Thus, in the case of EP-4 and EP-5, the presence HPc and BaTiO_3_ led to a decrease in the crosslink density, which decreased the *T_g_* values. In addition, the reduction in cross-linking density in the case of phosphorus containing thermosets could be due to the lower reactivity of phenolic groups of BPH.

Further discussion may be correlated with theoretical prediction of *T_g_*’s on binary mixtures, according to the Fox equation [39]:

(1) $$\frac{1}{T_{g}}=\frac{w_{1}}{T_{g,1}}+\frac{w_{2}}{T_{g,2}}$$

where *T_g_* represents the glass transition of the two-component systems, *w*_1_ and *w*_2_ are the weight fractions of the two components, and *T_g_*_,1_ and *T_g_*_,2_ are individual glass transition temperatures for each component.

Thus, for formulations containing BPH (Ep-1 to EP-5), the values obtained using Equation (1) for estimating the *T_g_* for plasticized glassy thermosets are higher than those obtained experimentally. For example, the calculated value for the EP-2 system is 186 °C, while the experimentally determined value is 158 °C. A possible explanation for these findings can be correlated with a decreased cross-linking density doubled by the plasticization effect that may occur in the composite systems. However, due to the extensive overlap of the absorption bands characteristic for ArPO_3_ groups (peak at 920 cm^−1^) and for epoxy groups (peak at 915 cm^−1^) in the current systems, this assumption is difficult to be confirmed rigorously. Figure S6 presents the FTIR spectrum of EP-2 containing a high concentration of BPH in the interval of 500–1000 cm^−1^. An absorption peak at around 915 cm^−1^ was evidenced, suggesting the presence of a low concentration or unreacted oxirane groups in the cured EP-2 thermoset. However, the intensity of this peak is very low.

**Figure 7:**
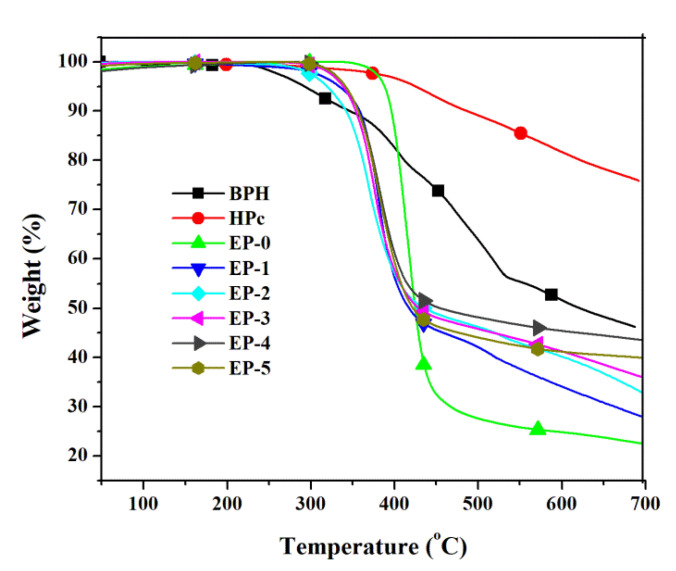
TG curves of epoxy thermosets, BPH, and HPc.

**Figure 8:**
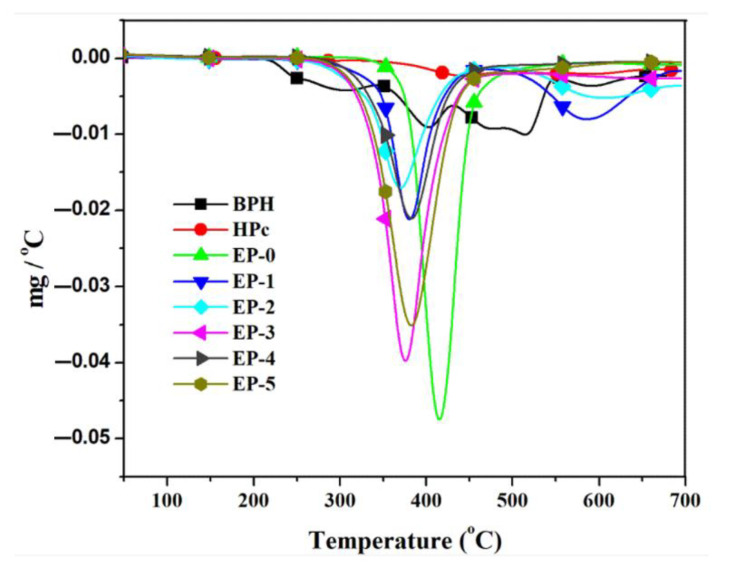
DTG curves of epoxy thermosets, BPH, and HPc.

The thermal stability of epoxy thermosets was evaluated by thermogravimetric analysis (TGA) and the main parameters (temperature at which the thermal degradation starts, T*_onset_*, temperature at which the mass loss is the highest, T*_max_*, and mass residue remaining after thermal degradation at 700 °C (char yield)) are summarized in Table 2. Typical TG and DTG thermograms of the samples are shown in Figure 7 and Figure 8, respectively.

As can be seen, BPH decomposed at lower temperatures in comparison with those of EP-0. However, the mass loss rate was lower, especially in the interval 550–700 °C, resulting in a high quantity of carbonaceous residue with relatively high thermal stability. On comparison with EP-0, epoxy thermosets containing BPH presented a decrease in the mass loss rate, especially in the temperature range of 450–700 °C, which led to higher values of char yields at 700 °C. The thermogravimetric curves of thermosets showed one step of decomposition. T*_onset_* was higher for the neat epoxy thermosets. The introduction in the thermoset structures of the segment coming from aromatic bisphenol BPH decreased the T*_onset_* values, especially in the case of the EP-2 containing the highest content of BPH. This is due to the lower initial decomposition temperature of BPH, which started to decompose at temperatures higher than 250 °C. T*_max_* values of the nanocomposites were in the temperature range of 368–412 °C. It can be observed that the maxima weight loss rates of the cured epoxy nanocomposites were lower than that of neat epoxy thermoset EP-0, EP-2 exhibiting the lowest value because of the higher content of BPH in the polymer matrix. The DTG curves showed that the decomposition rate of the epoxy thermosets decreased with increasing BPH content, which reveals the inhibition of thermal degradation of epoxy resin composites [33].

The heat resistance index T_HRI_ [27] was also calculated with Equation (2). This parameter presented in Table 2 indicates the ability of polymer composites to resist a heat flow.

(2) $$T_{\mathrm{HRI}}=0.49\cdot \left[T_{5\%}+0.6\cdot \left(T_{30\%}-T_{5\%}\right)\right]$$

T_5%_ is the temperature where 5 wt% of the weight was lost and T_30%_ is the corresponding decomposition temperature of 30% weight loss.

The highest values of T_HRI_ for epoxy thermosets were obtained for sample EP-3 containing BPH and HPc and samples EP-4 and Ep-5 containing BPH, HPc, and BaTiO_3_.

Char yield at 700 °C was in the range of 22.12–43.24 wt%. As expected, the lowest char yield value was exhibited by sample EP-0, not containing phosphorus atoms. The use of the bisphenol BPH as co-hardener considerably increased the char yield value; it increased further by increasing the BPH content. Thus, EP-2 showed a char yield of 31.20 wt%. High char yield value was achieved also for EP-3 containing BPH and HPc (35.81 wt%), suggesting that HPc additionally increased this parameter. The highest value of char yield was achieved for EP-4 containing the three additives, BPH, HPc, and BaTiO_3_. Further introspection on the char formation mechanism in the studied thermosets was realized by comparing the measured values of char residue at 700 °C with the calculated values derived from weighted summation of the values for individual components in the samples. Thus, for example, in the case of EP-2, the calculated value for char residue was 27.6 wt%. Since the values of char for EP-2 obtained from TGA was 31.20 wt%, the positive discrepancy (δ = +3.6 wt%) suggests that during the thermal treatment, supplemental chemical interactions between the individual components (EP-0 and BPH) took place, resulting in an enriched char yield. On the contrary, in the case of EP-5, the calculated value for char residue was 44.4 wt% compared to the measured one (39.86 wt%), revealing a negative discrepancy (δ = −3.54 wt%). Therefore, the presence of a higher content of BaTiO_3_ nanoparticles reduces considerably the formation of char in EP-5, which can be interpreted as a catalytic effect on the surface of the particles to degrade or interfere with the formation of the surrounding char network.

SEM images of the chars obtained by heating the samples up to 700 °C with the heating rate of 10 °C/min in nitrogen atmosphere are shown in Figure 4b. Char, a carbonaceous residue, results from the thermal degradation of the material being pyrolyzed. The char of phosphorus-free EP-0 was porous. In the case of EP-1 and especially EP-2, the charring properties have been improved; the chars were dense and compact, suggesting that they can better prevent the heat transfer between the flame zone and burning substrate [40,41]. Thus, the bisphenol BPH is an effective flame retardant for epoxy resin. The presence of both HPc and BPH as components in EP-3, EP-4, and EP-5 led to dense, compact, and homogeneous chars. In the case of EP-4 and EP-5, the particle of BaTiO_3_ can be seen incorporated in the char residues.

A mapping technique was used to investigate the atom distribution on the char surface of EP-3 and EP-4. Figure S8 and Figure 5b present the EDX mapping of EP-3 and EP-4 chars, respectively. Phosphorus and Cu atoms were observed on the char residue surface of EP-4, distributed relative uniform. In the case of EP-5, Ba and Ti atoms can be identified in the BaTiO_3_ particles incorporated in the char residues.

### 3.4. Microscale Combustion Calorimetry (MCC) Tests

**Figure 9:**
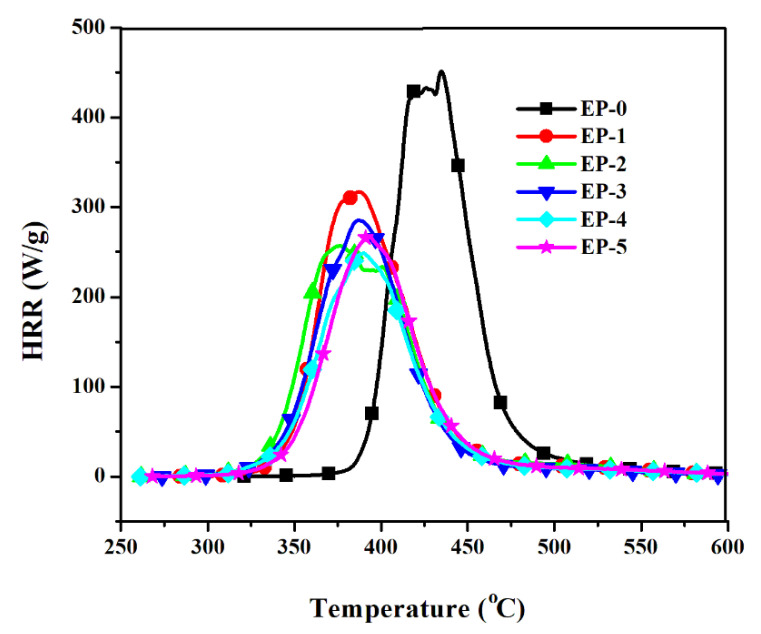
Heat release rates versus temperature for epoxy composites.

Data obtained from MCC analysis are summarized in Table 3. The results are shown comparatively in Figure 9 and Figure S9 where the dependencies of the heat release rate as a function of temperature and time, respectively, are presented.

As shown in Figure 9 and Figure S9, from the HRR curve of neat EP-0 it could be indicated that the HRR value rose very fast and reach a PHRR value of 451.68 W/g. The introduction of bisphenol BPH in the structure of the thermosets decreased the PHHR values 317.03 W/g for EP-1 and 257.23 W/g for EP-2. EP-4 and EP-5 also exhibited low values of PHRR. The lowest value of PHHR exhibited EP-4 containing phosphorus, hyperbranched phthalocyanines HPc and BaTiO_3_.

The HRC can be calculated from MCC tests and can be used to classify the flammability of the materials [42]. A low value indicates a low flammability of the samples [43]. The HRC of EP-0 was 776.03 J/(g*K). A substantial decrease in HRC values appeared by the introduction of bisphenol BPH (EP-1 and EP-2). The HRC value decreased further by the introduction of HPc in the case of EP-3 (HRC = 287.79 J/(g*K)). The lowest values of HRC were achieved for EP-4 and EP-5 containing BaTiO_3_, probably because of a lower concentration of epoxy resin in the system.

The THR value of the neat epoxy thermoset was 24.73 kJ/g. The THR of the samples containing segments coming from bisphenol BPH showed lower values. Thus, EP-2 exhibited a value of THR of 20.53 kJ/g. The lowest value was obtained for EP-4 containing HPc and BaTiO_3_ (THR = 17.51 kJ/g). The results show that the bisphenol, HPc, and BaTiO_3_ inhibited the thermal decomposition of epoxy thermosets.

The CY of the neat EP-0 was 13.96 wt%. A substantial increase in CY was observed for the samples EP-1 and EP-2 (24.65 wt% and 28.56%, respectively). The highest char yield was obtained for EP-4 (37.05 wt%). More residual char generated during pyrolysis, which inhibited the combustion of the epoxy thermosets and prevented the heat transfer to the matrix interior, proved the flame-retardant effect in the condensed phase. Similar conclusions were suggested by Zheng et al. in the study of flame retardancy for a hyperbranched phosphorus-containing copper phthalocyanine compound [33].

On comparing the results obtained from TGA and MCC measurements, although the heating rates of the analyses were quite different, the temperature dependence of HRR thermosets could be correlated with thermogravimetric analyses. Thus, in the case of EP-0, the main decomposition temperature took place in the temperature range of 380–500 °C. In the same range of temperature can be observed the increase of HRR having a high value of PHHR (451.68 W/g) at 438.87 °C. The introduction of BPH in epoxy thermosets modified the thermal decomposition process. The samples started to decompose at a lower temperature (323–356 °C) and the temperatures at which the mass loss is the highest exhibited lower values (368–376 °C). In the same time from MCC analyses was observed a decrease in T_PHHR_, in the interval 376.26–392.5 °C. An increase in char yield was observed, which correlates well with THR values, in the range of 17.5–21.41 kJ/g. According to TGA data, higher char yield results in a lower quantity of combustible gases and the formation of a protective layer (as was observed in SEM analyses) that can isolate the material, thus preventing the heat transfer and the transfer of combustible gases resulting during pyrolysis.

## 4. Conclusions

In the present work, DGEBA-based thermosets loaded with a bisphenol flame retardant containing DOPO and phenylphosphonate units, at various mass ratios of the raw materials, were developed and examined by means of FTIR, SEM, DSC, TGA, and MCC analysis. The scanning electron microscopy revealed a uniform distribution of BPH and HPc in the polymer matrix, while in the case of BaTiO_3_ nanoparticles, the presence of some agglomerations was highlighted. Epoxy thermosets exhibited high glass transition temperatures, in the range of 158–201 °C, and high decomposition temperatures. The incorporation of BPH and HPc into epoxy thermosets considerably increased the char yield at 700 °C, thus improving the characteristics of the carbonaceous residues. In addition, the reaction-to-fire characteristics in the series were discussed in relation to the presence of BPH, HPc, and BaTiO_3_ particles. The lower values of CY in comparison with those obtained by TGA are related to the specificity of the MCC technique that involves a fuel-generation process in anaerobic conditions at the burning surface where the flame consumes all of the available oxygen species evolved during the pyrolysis. The best flame resistance characteristics were achieved in the case of a sample containing all three components (BPH, HPc, and BaTiO_3_), as MCC analysis revealed (HRC and THR values lower than those of EP-0 by 67.7% and 30%, respectively, and CY value higher than that of EP-0 by 165%). Nevertheless, a higher content of BaTiO_3_ has a negative impact on the char formation mechanism.

## Figures and Tables

**Table 1:** Detailed data regarding the ingredients used in the preparation of the epoxy thermosets.

Sample	Epoxy Resin (g/Epoxy Equiv. *)	DDS (g)	Ratio of Reactive H DDS/Epoxy Equiv.	BPH (g)	Ratio of Reactive H BPH/Epoxy Equiv.	HPc (g)	BaTiO_3_ (g)	BPH (wt%)
EP-0	5/0.0265	1.64	0.996	-	-	-	-	-
EP-1	5/0.0265	1.54	0.937	0.77	0.0592	-	-	10.53
EP-2	5/0.0265	1.425	0.867	1.725	0.1328	-	-	21.16
EP-3	5/0.0265	1.525	0.928	0.887	0.0683	0.825	-	10.76
EP-4	5/0.0265	1.525	0.928	0.887	0.068	0.825	0.915	9.69
EP-5	5/0.0265	1.525	0.928	0.887	0.068	0.825	2.05	8.62

* EEW—Epoxy equivalent weight (EEW) is 0.53 equiv. per 100 g.

**Table 2:** Thermal properties of epoxy thermosets.

Sample	*T_g_* ^1^ (°C)	T_HRI_ ^2^ (°C)	T*_onset_* ^3^ (°C)	T*_max_* ^4^ (°C)	Char Yield at 700 °C (wt%)
EP-0	201	196	388	412	22.12
EP-1	170	177	356	376	27.50
EP-2	158	172	323	368	31.20
EP-3	159	195	338	374	35.81
EP-4	163	180	327	381	43.24
EP-5	161	179	337	380	39.86
BPH		196			46.23
HPc		304			75.22

^1^ Glass transition temperature (midpoint); ^2^ the heat resistance index temperature; ^3^ temperature at which the thermal degradation starts; ^4^ temperature at which the mass loss is the highest.

**Table 3:** Data obtained by MCC analysis for the epoxy thermosets.

Sample	CY ^1^ (wt%)	HRC ^2^ (J/(g*K))	THR ^3^ (kJ/g)	PHRR ^4^ (W/g)	T_PHRR_ ^5^ (°C)	Time ^6^ (s)
EP-0	13.96	776.03	24.73	451.68	434.87	115.50
EP-1	24.65	330.48	21.41	317.03	387.66	106.00
EP-2	28.56	323.59	20.53	257.23	376.26	116.50
EP-3	32.00	287.79	18.96	284.99	386.91	115.00
EP-4	37.05	250.52	17.51	248.62	390.10	130.50
EP-5	36.42	270.11	18.01	267.26	392.57	126.50

^1^ Char yield; ^2^ heat release capacity; ^3^ total heat release; ^4^ heat release peak; ^5^ temperature of heat release peak; ^6^ the time to attain heat release peak.

## Data Availability

The data that support the findings of this study are contained within the article and in the Supplementary Information.

## Supplementary Materials

The following supporting information can be downloaded at https://www.mdpi.com/article/10.3390/ma15217829/s1: Figure S1: FTIR spectrum of BPH; Figure S2: ^1^H NMR spectra of BPH; Figure S3: ^13^C NMR spectra of BPH; Figure S4: ^31^P NMR spectra of BPH; Figure S5: DSC curve of BPH; Figure S6: TG and DTG curves of BPH; Figure S7: EDX mapping of EP-3; Figure S8: EDX mapping of EP-3 char surface; Figure S9: Heat release rates versus time for epoxy composites.
