# Supplementary material for: Thermal Properties and Flammability Characteristics of a Series of DGEBA-Based Thermosets Loaded with a Novel Bisphenol Containing DOPO and Phenylphosphonate Units
Source: Materials (Basel). 2022 Nov 6;15(21):7829. doi: 10.3390/ma15217829 (PMC9657289; doi:10.3390/ma15217829)
Supplement: Supplementary file 1 [file materials-15-07829-s001.zip › materials-1955426-supplementary.pdf]

materials

Supplementary Materials

Thermal Properties and Flammability Characteristics of a Series of DGEBA-Based Thermosets Loaded with a Novel Bisphenol Containing DOPO and Phenylphosphonate Units

Corneliu Hamciuc [1,*], Tăchiță Vlad-Bubulac [1,*], Diana Serbezeanu [1], Ana-Maria Macsim [1], Gabriela Lisa [2], Ion Anghel [3] and Ioana-Emilia Şofran [3]

[1] Department of Polycondensation and Thermally Stable Polymers, "Petru Poni" Institute of Macromolecular Chemistry, 41A, Grigore Ghica Voda Alley, 700487 Iasi, Romania
[2] Department of Chemical Engineering, Faculty of Chemical Engineering and Environmental Protection, "Gheorghe Asachi" Technical University of Iasi, Bd. Mangeron 73, 700050 Iasi, Romania
[3] Fire Officers Faculty, Police Academy "Alexandru Ioan Cuza", Morarilor Str. 3, Sector 2, 022451 Bucharest, Romania
* Correspondence: chamciuc@icmpp.ro (C.H.); tvladb@icmpp.ro (T.V.-B.)

Citation: Hamciuc, C.; Vlad-Bubulac, T.; Serbezeanu, D.; Macsim, A.-M.; Lisa, G.; Anghel, I.; Şofran, I.-E. Thermal Properties and Flammability Characteristics of a Series of DGEBA-Based Thermosets Loaded with a Novel Bisphenol Containing DOPO and Phenylphosphonate Units. *Materials* **2022**, *15*, 7829. https://doi.org/10.3390/ma15217829

Academic Editors: Gisbert Riess and Florian Arbeiter

Received: 21 September 2022
Accepted: 4 November 2022
Published: 6 November 2022

Publisher's Note: MDPI stays neutral with regard to jurisdictional claims in published maps and institutional affiliations.

Copyright: © 2022 by the authors. Licensee MDPI, Basel, Switzerland. This article is an open access article distributed under the terms and conditions of the Creative Commons Attribution (CC BY) license (https://creativecommons.org/licenses/by/4.0/).

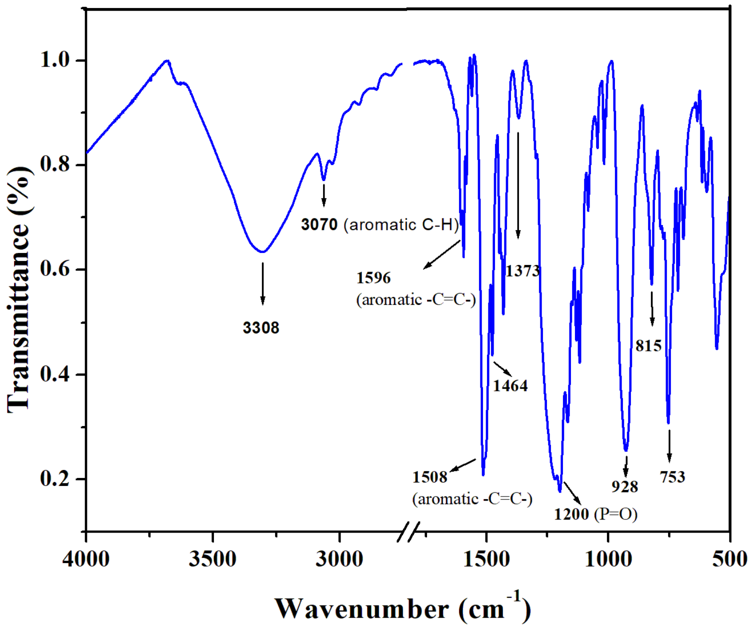

Figure S1. FTIR spectrum of BPH.

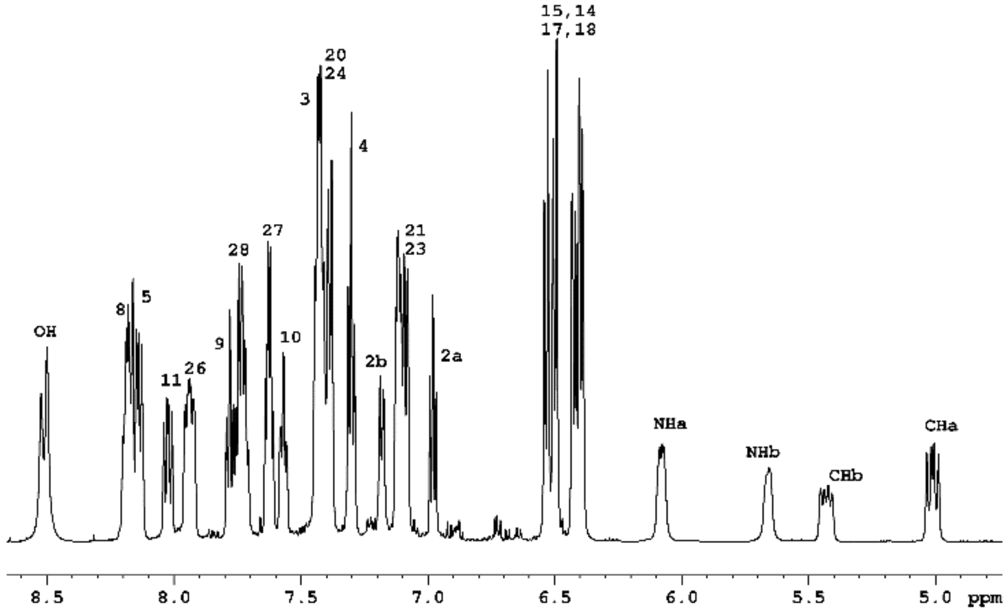

Figure S2. ^{1}H NMR spectra of BPH.

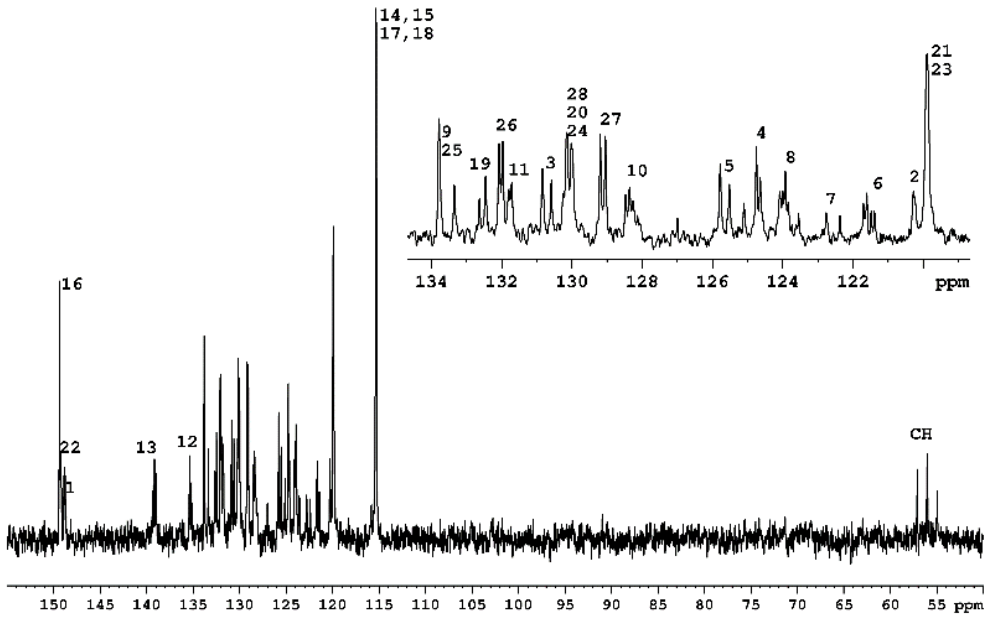

Figure S3. ^{13}C NMR spectra of BPH.

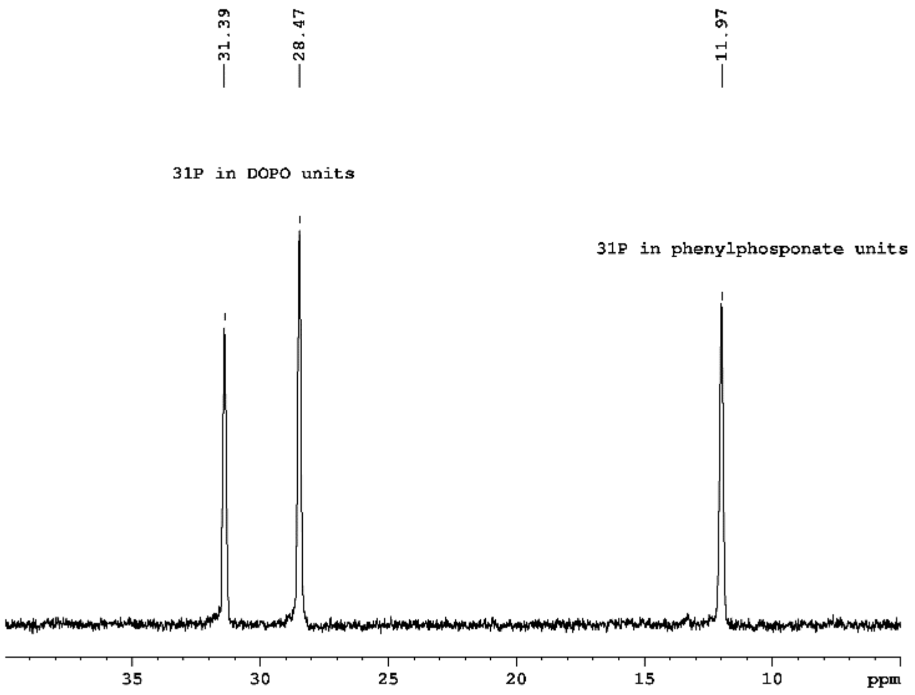

Figure S4. ^{31}P NMR spectra of BPH.

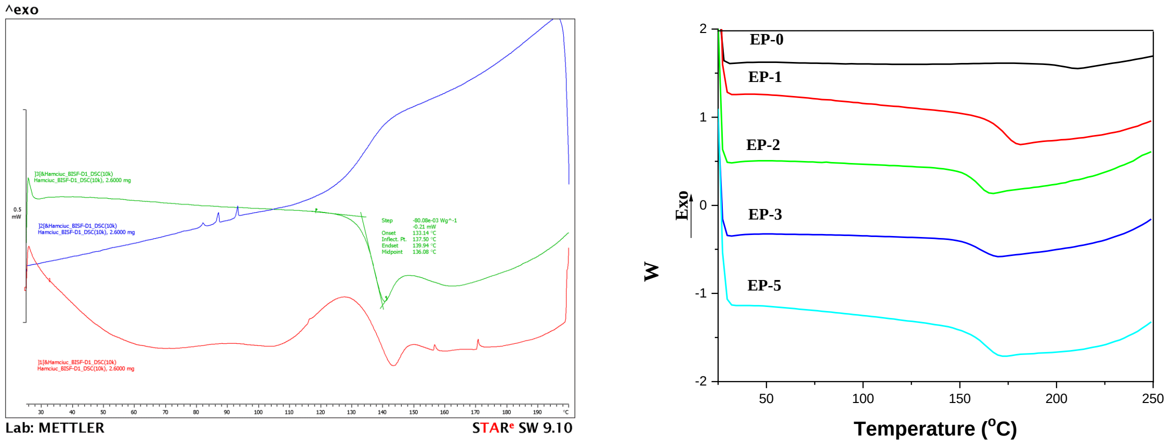

(**a**) (**b**)

Figure S5. (a) DSC curves of BPH (three scans); (b) DSC curves of epoxy thermosets.

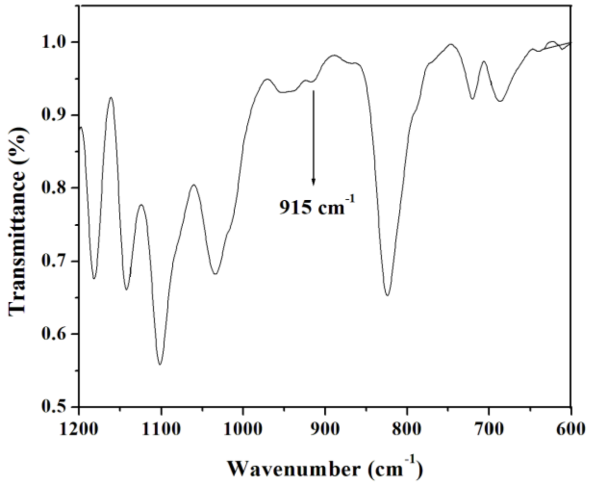

Figure S6. FTIR spectrum of EP-2 in the interval 1200-500 cm^{-1}.

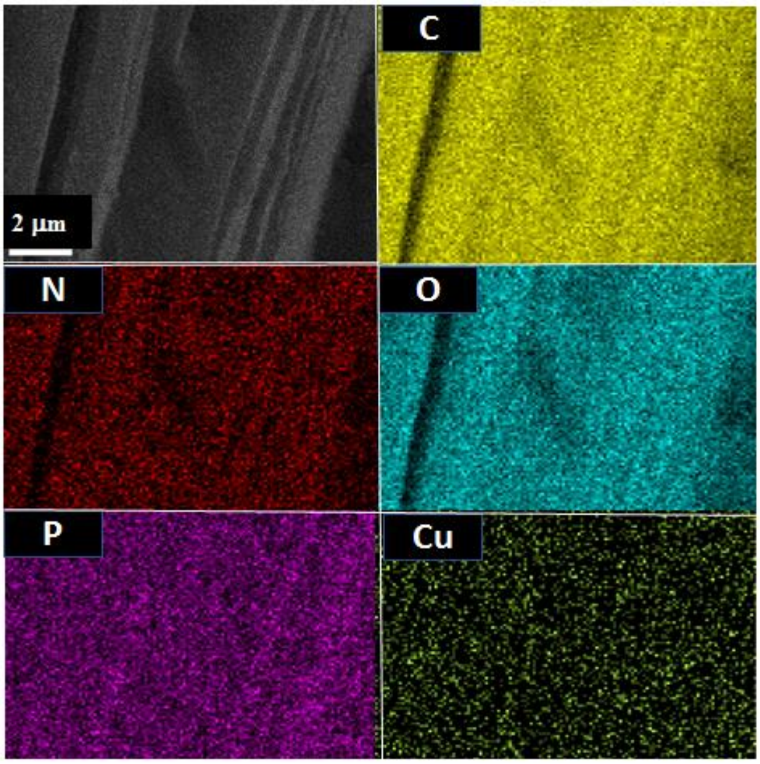

Figure S7. EDX mapping of EP-3.

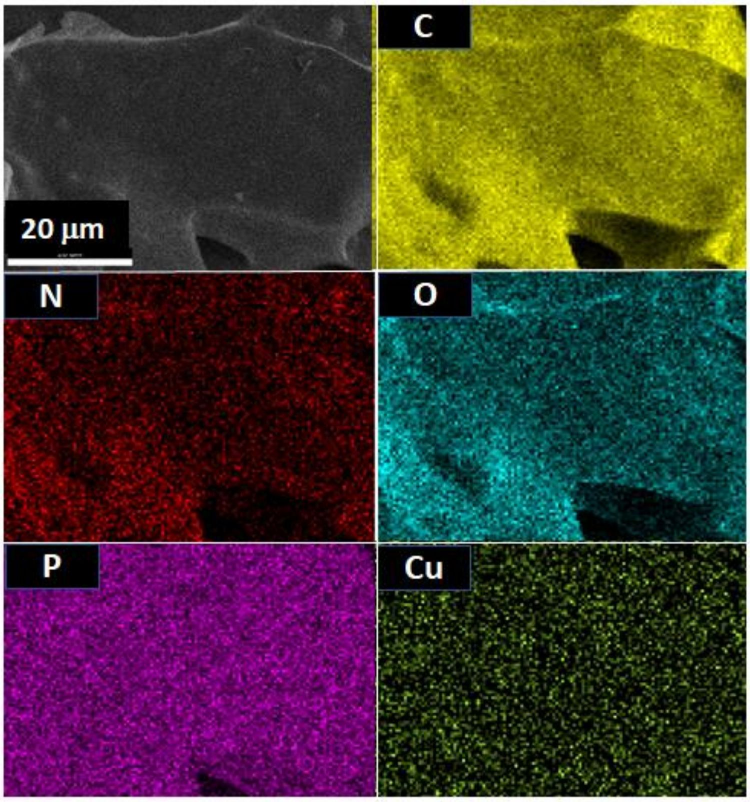

Figure S8. EDX mapping of EP-3 char surface.

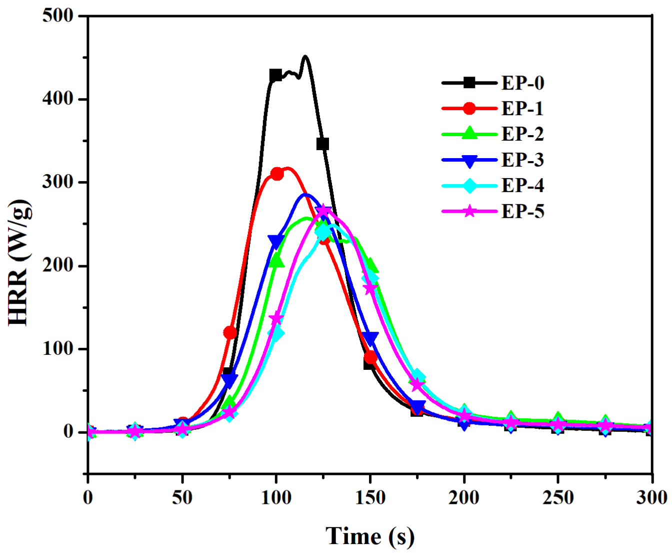

Figure S9. Heat release rates versus time for epoxy composites.
